# BMI and Risk of Serious Upper Body Injury Following Motor Vehicle Crashes: Concordance of Real-World and Computer-Simulated Observations

**DOI:** 10.1371/journal.pmed.1000250

**Published:** 2010-03-30

**Authors:** Shankuan Zhu, Jong-Eun Kim, Xiaoguang Ma, Alan Shih, Purushottam W. Laud, Frank Pintar, Wei Shen, Steven B. Heymsfield, David B. Allison

**Affiliations:** 1 Injury Research Center, Medical College of Wisconsin, Milwaukee, Wisconsin, United States of America; 2 Injury Control Research Center, and Obesity and Body Composition Research Center, Zhejiang University School of Public Health, Hangzhou, China; 3 Department of Mechanical Engineering, University of Alabama at Birmingham, Birmingham, Alabama, United States of America; 4 Department of Population Health, Division of Biostatistics, Medical College of Wisconsin, Milwaukee, Wisconsin, United States of America; 5 Neurosurgery Neuroscience Lab, Medical College of Wisconsin, Milwaukee, Wisconsin, United States of America; 6 Obesity Research Center, St. Luke's Roosevelt Hospital and Institute of Human Nutrition, Columbia University College of Physicians and Surgeons, New York, New York, United States of America; 7 Center for Scientific Affairs, Merck & Co., Rahway, New Jersey, United States of America; 8 Department of Biostatistics, and Nutrition and Obesity Research Center, University of Alabama at Birmingham, Alabama, United States of America; Research Center INSERM U897 “Epidémiologie et Biostatistiques”, France

## Abstract

**Background:**

Men tend to have more upper body mass and fat than women, a physical characteristic that may predispose them to severe motor vehicle crash (MVC) injuries, particularly in certain body regions. This study examined MVC-related regional body injury and its association with the presence of driver obesity using both real-world data and computer crash simulation.

**Methods and Findings:**

Real-world data were from the 2001 to 2005 National Automotive Sampling System Crashworthiness Data System. A total of 10,941 drivers who were aged 18 years or older involved in frontal collision crashes were eligible for the study. Sex-specific logistic regression models were developed to analyze the associations between MVC injury and the presence of driver obesity. In order to confirm the findings from real-world data, computer models of obese subjects were constructed and crash simulations were performed. According to real-world data, obese men had a substantially higher risk of injury, especially serious injury, to the upper body regions including head, face, thorax, and spine than normal weight men (all *p<*0.05). A *U*-shaped relation was found between body mass index (BMI) and serious injury in the abdominal region for both men and women (*p<*0.05 for both BMI and BMI^2^). In the high-BMI range, men were more likely to be seriously injured than were women for all body regions except the extremities and abdominal region (all *p<*0.05 for interaction between BMI and sex). The findings from the computer simulation were generally consistent with the real-world results in the present study.

**Conclusions:**

Obese men endured a much higher risk of injury to upper body regions during MVCs. This higher risk may be attributed to differences in body shape, fat distribution, and center of gravity between obese and normal-weight subjects, and between men and women.

*Please see later in the article for the Editors' Summary*

## Abstract 2

Shankuan Zhu and colleagues use computer crash simulations, as well as real-world data, to evaluate whether driver obesity is associated with greater risk of body injury in motor vehicle crashes.

## Editors' Summary

**Background:**

Worldwide, accidents involving motor vehicles kill 1.2 million people and injure as many as 50 million people every year. Collisions between motor vehicles, between vehicles and stationary objects, or between vehicles and pedestrians are responsible for one in 50 deaths and are the 11th leading cause of death globally. Many factors contribute to the risk of motor traffic accidents and the likelihood of subsequent injury or death. These risk factors include vehicle design, vehicle speeds, road design, driver impairment through, for example, alcohol use, and other driver characteristics such as age. Faced with an ever-increasing death toll on their roads, many countries have introduced lower speed limits, mandatory seat belt use, and greater penalties for drunk driving to reduce the carnage. Road design and traffic management initiatives have also been introduced to try to reduce the incidence of road traffic accidents and cars now include many features that provide protection in crashes for their occupants such as airbags and crumple zones.

**Why Was This Study Done?:**

Although these measures have reduced the number of crashes and casualties, a better understanding of the risk factors associated with motor vehicle crashes is needed to deal with this important public-health problem. Another major public-health problem is obesity—having excess body fat. Obesity increases the risk of heart disease and diabetes but also contributes to the severity of motor vehicle crash injuries. Men with a high body mass index (an individual's weight in kilograms divided by height in meters squared; a BMI of 30 or more indicates obesity) have a higher risk of death after a motor vehicle accident than men with a normal BMI (18.5–24.9). This association between death and obesity is not seen in women, however, possibly because men and women accumulate fat on different parts of their body and the resultant difference in body shape could affect how male and female bodies move during traffic collisions and how much protection existing car safety features afford them. In this study, therefore, the researchers investigated how driver obesity affects the risk of serious injuries in different parts of the body following real and simulated motor vehicle crashes in men and women.

**What Did the Researchers Do and Find?:**

The researchers extracted data about injuries and BMIs for nearly 11,000 adult men and women who were involved in a frontal motor vehicle collision between 2001 and 2005 from the Crashworthiness Data System of the US National Automotive Sampling System. They then used detailed statistical methods to look for associations between specific injuries and driver obesity. The researchers also constructed computer models of obese drivers and subjected these models to simulated crashes. Their analysis of the real-world data showed that obese men had a substantially higher risk of injury to the upper body (the head, face, chest, and spine) than men with a normal weight. Serious injury in the abdominal region was most likely at low and high BMIs for both men and women. Finally, obese men were more likely to be seriously injured than obese women for all body regions except the extremities and the abdominal region. The researchers' computer simulations confirmed many of these real-world findings.

**What Do These Findings Mean?:**

These findings suggest that obese men have a higher risk of injury, particularly to their upper body, from motor vehicle crashes than men with a normal body weight or than obese women. The researchers suggest that this higher risk may be attributed to differences in body shape, fat distribution, and center of gravity between obese and normal weight individuals and between men and women. These findings, although limited by missing data, suggest that motor vehicle safety features should be adjusted to take into account the ongoing obesity epidemic. Currently, two-thirds of people in the US are overweight or obese, yet a crash test dummy with a normal BMI is still used during the design of car cabins. Finally, although more studies are needed to understand the biomechanical responses of the human body during vehicle collisions, the findings in this study could aid the identification of groups of people at particularly high risk of injury or death on the roads who could then be helped to reduce their risk.

**Additional Information:**

Please access these Web sites via the online version of this summary at http://dx.doi.org/10.1371/journal.pmed.1000250.

- Wikipedia has a page on traffic collision (note that Wikipedia is a free online encyclopedia that anyone can edit; available in several languages)
- The World Health Organization has information about road traffic injuries as a public-health problem; its World report on road traffic injury prevention is available in several languages
- The US Centers for Disease Control and Prevention provides detailed information about overweight and obesity (in several languages)
- MedlinePlus provides links to further resources about obesity (in English and Spanish)
- The US National Automotive Sampling System Crashworthiness Data System contains detailed data on thousands of US motor vehicle crashes

## Introduction

Motor vehicle accidents are the leading cause of injury-related death in the United States. In 2005, approximately 45,000 persons died and more than 3 million persons were injured in motor vehicle accidents in the United States [1]. The estimated economic cost of motor vehicle accidents in 2005 was approximately 230 billion dollars [2]. Establishing the mechanisms, risk factors, and potential preventive strategies for motor vehicle accidents is a major goal of public health efforts.

A large body mass with excess adiposity may contribute to motor vehicle crash (MVC) injuries in several ways, although little is known about the relation between obesity and the risk of injury. Our previous study identified sex differences in the relations between MVC fatality and body mass index (BMI) [3]. Men had a higher risk of death at both the high and low ends of the BMI continuum, whereas this pattern was not present in women. The cause of this differential association between BMI and MVC-related death between men and women is presently unknown.

One potential mechanism for sex differences in the risk of injury in MVCs is that body shape and fat distribution are important markers of mortality and injury risk. Fat distribution differs between men and women, including the amount and proportion of subcutaneous and visceral fat along with related waist and hip girths. The gynoid pattern with lower body adiposity, as defined in 1947 by Vague [4], is most frequently observed in women, whereas upper body adiposity is typical of men [5]–[7]. Does the sex difference in body shape and fat distribution affect injury severity and pattern during an MVC?

We investigated the association between obesity as defined by BMI and regional injuries during MVCs on the basis of both real-world nationally representative data and simulated crash data using computational models of obese occupants. The aim of this study was to explore the potential contribution of sex-specific differences in body shape towards the pattern and severity of injuries. We hypothesized that the risk of regional body injuries would increase with BMI in both men and women and that this weight-related increase would be more significant among men.

## Methods

### Real-World Data Analysis

#### Study population

We used data from the Crashworthiness Data System (CDS) of the National Automotive Sampling System (NASS). The NASS CDS is a nationally representative database containing information on accidents, vehicles, and occupants [8],[9].

We initially included as potential subjects 13,710 passenger car or truck drivers aged 18 y and older involved in frontal-collision MVCs from 2001 to 2005. From this cohort, 2,547 subjects were excluded for the following reasons: pregnant female driver (*n = *111), sex unknown (*n = *8), death from other reasons (*n = *51), death with injury unknown (*n = *51), treatment status unknown (*n = *242), and lack of information on height or weight (*n = *2,287). In addition, 222 subjects with a BMI (in kg/m^2^) less than 17.3 (*n = *111) or greater than 46.0 (*n = *111)—the points that approximately corresponded to the 1st and 99th percentiles of the BMI distribution in the database—were excluded to avoid potential bias that might have been caused by measurement or input errors. Therefore, a total of 10,941 subjects were included in the analysis, of whom 61.4% were men and 38.6% women.

#### Outcome definition

The primary outcome variable was drivers' regional body injury as measured by using the Abbreviated Injury Scale (AIS), the most widely used anatomic scale for rating severity of injuries. The body regions included the head, face, thorax, abdomen, spine, upper extremities, and lower extremities. Having a regional body injury was defined as an AIS >0 for any of the evaluated body regions, whereas having a serious regional body injury was defined as an AIS >2 [10].

#### Statistical analysis

Data are presented as means (for continuous variables) or as percentages (for categorical variables) with 95% confidence intervals (CIs). The characteristics of drivers, collisions, and the environment were compared by using *t*-tests (for continuous variables) or chi-square tests (for categorical variables) between the sexes. Logistic regression was used to examine the relations between each regional body injury with BMI (BMI and BMI^2^) after adjustment for potential confounding factors for male and female drivers separately. Before building sex-specific regression models, the data for men and women were pooled, and the interaction between sex and BMI (BMI and BMI^2^) was tested by using the same logistic regression model to examine whether the interaction term was significant. The potential confounding factors included in the regression model were age, race, type of vehicle, vehicle age and weight, alcohol or drug use, seatbelt use, airbag deployment, ejection, rollover, number of vehicles involved, road speed limit, light, and weather conditions. Sensitivity analysis for every factor was tested by including or excluding it in the model to detect its impact on the models and results. Seat belt use is an important variable because unbelted drivers are at much higher risk of injury and could therefore skew the results. Thus, models were estimated for belted, unbelted, and pooled drivers to detect any potential skewing of the results.

Two logistic regression models were developed: (1) the all subjects model, which included all subjects, and (2) the change of velocity model (ΔV model), which included information on the change of velocity during the crash (ΔV, km/h). Approximately 44% of the subjects had no ΔV information in the NASS CDS datasets. ΔV was measured by NHTSA as part of the NASS data collection process using a computer program (WinSMASH, National Highway Traffic Safety Administration, Washington [D.C.]) that reconstructs a single 2-D vehicle-to-vehicle impact or a vehicle-to-large-object impact that resembles a barrier collision [8]. The characteristics of the subjects with and without ΔV information were compared. In addition, the relations between BMI and regional body injuries between subjects with and without ΔV information were examined by testing the interaction terms between BMI and the ΔV group indicator by the same logistic regression model.

To produce nationally representative estimates, all analyses incorporated sampling weights. Weighted means, percentages, odds ratios (ORs), and standard errors (SEs) were calculated by using Stata software (Version 10.0, Stata Corp.) to adjust for the complex NASS CDS sampling design. Statistical significance was set at *p<*0.05 (two-tailed). Since there were a large number of regression tests in present study, a conservative approach would be to apply the Bonferroni correction. However, following the guidelines by Bailar and Mosteller [11], we chose not to apply the Bonferroni correction per se, but rather reported the per test alpha level the Bonferroni correction would require (i.e., α is set as 0.05 divided by the number of tests, 0.05/168 = 0.0003). Therefore, we referred to each *p*-value as “not statistically significant” if *p*≥0.05, as “significant at the uncorrected nominal 0.05 level” if *p<*0.05, and as “significant even at the Bonferroni corrected level” if *p<*0.0003.

### Computer Modeling and Crash Simulation

Mathematical Dynamic Models, MADYMO version 7.0 (TNO, The Netherlands), was used for model simulation of a vehicle frontal impact tests. Male (BMI 30 and 35, height 1.77 m) and female (BMI 30 and 35, height 1.52 m) obese dummy models were developed on the basis of the well-validated, widely used, and computationally efficient MADYMO hybrid III dummy models: 50th percentile male (BMI 25, height 1.77 m) and 5th percentile female (BMI 22, height 1.52 m), respectively.

To take account of subcutaneous adipose tissue of obese subjects into the computer models, mask files of adipose tissue (color-coded for each anatomical region) acquired from grey-scale MRI datasets were utilized in this study [12],[13]. The exempt status of using the existing de-identified datasets for reconstruction was reviewed and approved by the Institutional Review Board of St. Luke's-Roosevelt Hospital. Each dataset consists of ∼43 mask files of adipose tissue generated from DICOM (Digital Imaging and Communications in Medicine) images with a 256 by 256 pixel resolution for each slice. A specialized computer tool was developed to reconstruct the adipose tissue regions into a 3-D geometry by discrete points in a triangulated manner. On the basis of the geometry data, finite element models for torso subcutaneous adipose tissue were developed. The torso adipose tissue model, which has the hyperelastic properties [14] and density (900 kg/m^3^) [15] of human subcutaneous adipose tissue, was then integrated into the standard MADYMO dummy models to represent obese occupants. The inertial parameters and sizes of limbs were increased as BMI increased.

The standard and obese dummy models were validated against experimental data from a set of sled tests with postmortem human surrogates (PMHS) [16]. The averaged test data from five nonobese males (mean height 1.77 m, weight 63.5 kg, BMI 20.3) and three obese subjects (mean height 1.79 m, weight 128.0 kg, BMI 40.1) were used to validate the standard male (BMI 25) and obese male (BMI 35) dummy models, respectively. The dummy models were seated in the rear seat with a force-limiting, pre-tensioning seatbelt system. The specific characteristics of the restraint system and crash pulse used in the PMHS tests were replicated in MADYMO model simulation to compare kinematics and trajectories.

Once the models were validated, they were used to examine the risk of injury to drivers during frontal crashes. A baseline configuration (referred to as Case 1) of the occupant restraint system is the following. The seat belt was equipped with buckle and retractor pre-tensioners and a load limiter with a maximum force of 4 kN. A crash deceleration pulse from a full rigid frontal barrier impact (ΔV = 56 km/h) [17] and a mass flow rate for airbag deployment [18] from literature were used. The seat belt webbing elongation was set to 10% at 10 kN force. Case studies were performed to consider a variety of restraint systems and crash pulses.

Case 2: No pre-tensioners and load limiter were equipped.

Case 3: The mass flow rate of the airbag was depowered by about 20%.

Case 4: The steering wheel angle was tilted 10 degrees downward.

Case 5: The crash deceleration pulse was scaled up to 1.2 times.

From the MADYMO simulation results, the following four regional body injuries were measured. The head injury criterion (HIC) is a measure of the likelihood of head injury based on acceleration at the center of gravity of the dummy's head. The neck injury criterion, called Nij, proposes critical limits for all four possible modes of neck loading: tension or compression combined with either flexion (forward) or extension (rearward) bending moment. The chest acceleration and chest deflection are used for thoracic injury criteria. The lower extremity criterion (LEC) is a measure of femur load for tolerance of leg injury. The details of these injury criteria can be found in literature [19]. No criteria for the face, spine, abdomen, and upper extremity, however, were specified.

## Results

The characteristics of the drivers, crashes, and environment of the crash by sex are shown in Table 1. Male drivers had on average higher mean BMI than female drivers. A greater proportion of female drivers than male drivers were driving passenger cars, were wearing seatbelts, and were in vehicles in which an airbag deployed. Female drivers were also driving relatively newer cars. Male drivers were driving vehicles with a higher vehicle weight and ΔV during the crash and were more likely to be involved in crashes involving alcohol use, ejection, and rollover. Male drivers were also more likely to have single-vehicle collisions than were female drivers. More male drivers had MVCs on roads with speed limits of 80 to 105 km/h, whereas more female drivers had crashes on roads with speed limits of 48 to 80 km/h. In addition, male drivers were involved in MVCs more frequently at night than were female drivers.

**Table 1:** Sample characteristics and injury outcomes, by sex.

Characteristics and Injury Outcomes	Male	Female	Pool
**Sample size**	6,715	4,226	10,941
**Weighted size**	3,020,809	2,170,622	5,191,431
**Driver characteristics, mean (95% CI) or % (95% CI)**			
Age, y	36.5 (35.7––37.2)	38.2 (36.4–40.0)*	37.2 (36.3–38.1)
Height, cm	178.6 (177.5–179.7)	164.3 (163.7–164.9)***	172.6 (171.9–173.3)
Weight, kg	84.9 (83.3–86.4)	68.4 (66.6–70.3)***	78.0 (76.7–79.3)
BMI, kg/m^2^	26.6 (26.3–26.8)	25.3 (24.7–25.9)***	26.1 (25.8–26.3)
Race, %			
White	60.7 (52.5–68.4)	62.6 (56.6–68.2)	61.6 (54.8–67.8)
Black	14.8 (9.8–21.8)	14.5 (10.2–20.1)	14.7 (11.1–19.2)
Hispanic	8.5 (4.6–15.1)	8.0 (3.8–16.1)	8.3 (4.7–14.2)
Other	3.6 (1.9–6.8)	2.6 (1.3–5.1)	3.2 (1.7–5.9)
Unknown	12.4 (7.4–20.0)	12.4 (8.9–16.9)	12.4 (8.1–18.4)
**Outcome variables, % (95% CI)**			
Max AIS			
Head			
Injured (Max AIS >0)	8.4 (5.3–13.3)	6.7 (3.6–12.0)*	7.7 (4.6–12.6)
Seriously injured (Max AIS >2)	0.9 (0.5–1.8)	0.3 (0.2–0.4)***	0.7 (0.4–1.1)
Face			
Injured (Max AIS >0)	14.2 (11.3–17.6)	11.7 (8.7–15.4)	13.1 (11.3–15.1)
Seriously injured (Max AIS >2)	0.2 (0.1–0.4)	0.0 (0.0–0.1)***	0.1 (0.1–0.2)
Thorax			
Injured (Max AIS >0)	9.8 (7.3–13.2)	18.8 (14.4–24.2)***	13.6 (10.2–17.8)
Seriously injured (Max AIS >2)	1.3 (0.7–2.3)	0.9 (0.5–1.4)*	1.1 (0.7–1.8)
Abdomen			
Injured (Max AIS >0)	4.1 (2.2–7.6)	5.1 (3.9–6.8)	4.5 (3.2–6.4)
Seriously injured (Max AIS >2)	0.3 (0.2–0.4)	0.2 (0.1–0.5)	0.3 (0.2–0.4)
Spine			
Injured (Max AIS >0)	9.1 (5.4–15.0)	14.5 (8.5–23.6)***	11.3 (6.7–18.6)
Seriously injured (Max AIS >2)	0.3 (0.2–0.6)	0.2 (0.1–0.4)**	0.3 (0.1–0.5)
Upper extremity			
Injured (Max AIS >0)	18.5 (15.2–22.4)	27.4 (24.0–31.0)***	22.2 (20.1–24.5)
Seriously injured (Max AIS >2)	0.4 (0.2–0.7)	0.6 (0.4–0.9)	0.5 (0.3–0.7)
Lower extremity			
Injured (Max AIS >0)	15.3 (12.4–18.7)	23.1 (20.7–25.7)***	18.6 (16.6–20.7)
Seriously injured (Max AIS >2)	1.2 (0.9–1.7)	0.7 (0.6–0.9)**	1.0 (0.8–1.3)
**Crash characteristics, mean (95% CI) or % (95% CI)**			
Type of vehicle, %			
Passenger	62.5 (59.4–65.5)	72.3 (66.2–77.6)***	66.6 (62.9–70.1)
Truck	37.5 (34.5–40.6)	27.7 (22.4–33.8)	33.4 (29.9–37.1)
Vehicle age, y	7.8 (7.2–8.3)	6.5 (5.8–7.1)***	7.2 (6.7–7.8)
Curb weight, kg	1,522.4 (1471.2–1573.4)	1,437.1 (1405.0–1468.9)***	1,486.0 (1448.1–1524.3)
Total ΔV, km/h	21.4 (20.8–22.0)	20.2 (19.4–21.1)***	20.9 (20.3–21.6)
Alcohol use, %			
Yes	12.5 (9.8–16.0)	6.4 (3.2–12.7)**	10.0 (7.1–13.8)
No	76.8 (70.8–82.0)	85.1 (79.8–89.3)	80.3 (74.7–84.9)
Unknown	10.6 (7.7–14.6)	8.5 (5.2–13.4)	9.7 (6.8–13.7)
Drug use, %			
Yes	3.6 (2.5–5.2)	4.2 (1.7–10.4)	3.9 (2.1–7.1)
No	77.1 (66.9–84.9)	80.7 (70.8–87.9)	78.6 (68.5–86.1)
Unknown	19.3 (11.9–29.8)	15.1 (7.8–27.2)	17.5 (10.2–28.5)
Seat belt use, %			
Yes	76.1 (72.7–79.3)	81.5 (78.2–84.4)***	78.4 (75.4–81.1)
No	17.7 (15.1–20.7)	13.7 (10.9–17.1)	16.1 (13.6–18.8)
Unknown	6.1 (4.5–8.3)	4.8 (3.8–6.1)	5.6 (4.5–7.0)
Air bag deployed, %			
Yes	32.2 (28.7–35.8)	37.8 (33.4–42.5)***	34.5 (31.1–38.2)
No	33.7 (30.6–37.0)	37.8 (34.8–41.0)	35.4 (33.4–37.6)
Not equipped	31.3 (27.1–36.0)	20.9 (16.4–26.1)	27.0 (23.1–31.2)
Unknown	2.8 (1.9–4.1)	3.5 (2.2–5.5)	3.1 (2.1–4.5)
Ejection, %			
Yes	1.0 (0.7–1.4)	0.4 (0.2–0.7)***	0.7 (0.5–1.1)
No	98.8 (98.4–99.0)	99.1 (97.4–99.7)	98.9 (98.2–99.3)
Unknown	0.3 (0.2–0.5)	0.6 (0.1–2.5)	0.4 (0.2–1.1)
Rollover, %			
Yes	5.8 (4.4–7.7)	3.8 (2.1–6.6)	5.0 (3.6–6.7)
No	93.9 (91.8–95.5)	95.7 (93.2–97.4)	94.7 (92.8–96.0)
Unknown	0.3 (0.1–0.7)	0.5 (0.2–1.7)	0.4 (0.2–1.0)
No. involved vehicles, %			
Single	36.4 (31.5–41.6)	30.2 (25.8–34.9)***	33.8 (29.6–38.3)
Two or more	62.3 (57.4–67.0)	68.7 (64.7–72.6)	65.0 (60.9–68.9)
Unknown	1.3 (0.6–2.8)	1.1 (0.4–3.3)	1.2 (0.6–2.4)
**Environment characteristics, % (95% CI)**			
Speed limit, %			
≤48 km/h	20.0 (14.6–26.6)	19.8 (13.4–28.3)***	19.9 (14.3–27.1)
>48 to ≤80 km/h	47.4 (40.3–54.7)	54.1 (45.1–62.9)	50.2 (42.7–57.8)
>80 to ≤105 km/h	24.4 (19.2–30.5)	18.2 (15.8–20.8)	21.8 (18.2–26.0)
>105 km/h	7.5 (3.8–14.0)	7.3 (4.2–12.4)	7.4 (4.0–13.3)
Unknown	0.7 (0.4–1.2)	0.5 (0.3–1.0)	0.6 (0.4–1.0)
Light condition, %			
Daylight	61.2 (57.5–64.7)	71.8 (68.3–75.1)***	65.6 (62.7–68.5)
Dark	14.0 (10.8–17.9)	10.2 (7.4–13.9)	12.4 (9.6–15.9)
Dark, but lighted	19.0 (16.4–21.9)	15.0 (12.5–17.8)	17.3 (15.8–19.0)
Others	5.7 (4.7–7.0)	2.9 (1.7–5.1)	4.6 (3.6–5.7)
Unknown	0.1 (0.0–0.5)	0.2 (0.0–1.2)	0.1 (0.0–0.8)
Weather, %			
No adverse conditions	82.3 (79.1–85.0)	82.0 (74.2–87.8)	82.2 (78.5–85.3)
Adverse conditions	17.6 (14.9–20.7)	17.7 (11.9–25.5)	17.6 (14.5–21.3)
Unknown	0.2 (0.0–0.8)	0.3 (0.2–0.6)	0.2 (0.1–0.6)

Comparisons between men and women were made with weighted *t*-test for continuous variables and chi-square test for categorical variables.

*, *p<*0.10.

**, *p<*0.05.

***, *p<*0.01.

Except for injuries to the head, face, and abdomen, the percentages of injury (injured versus not injured) to the thorax, spine, and upper and lower extremities were all higher for women than for men. However, the percentages of serious injury (seriously injured versus not seriously injured) to all body regions except for the thorax, abdomen, and upper extremity were greater for men than for women. Shown in Table 2 are the coefficients derived from the logistic regression of BMI (BMI and BMI^2^) for each regional body injury measured by each of two outcomes, injured versus not injured (left) and seriously injured versus not seriously injured (right), in the all subjects model (upper) and in the ΔV model (lower). In the all subjects model, the sex difference was present only for injuries to the lower extremities for the injured versus not injured outcome. In the ΔV model, regional body injuries to the face, thorax, and abdomen differed significantly between male and female drivers for the injured versus not injured outcome and regional body injuries to the head, thorax, and spine differed significantly between male and female drivers for the seriously injured versus not seriously injured outcome.

**Table 2:** Logistic regression coefficients of BMI for regional body injury severity, by sex.

Body Region	Injured Versus Not Injured^a^			Seriously Injured Versus Not Seriously Injured^a^		
	Male (Coefficient)^b^	Female (Coefficient)^b^	Pooled (*p-*Value)^c^	Male (Coefficient)^b^	Female (Coefficient)^b^	Pooled (*p*-Value)^c^
**All subjects model**						
Head						
Linear (BMI)	0.0502**	−0.0141	0.077	0.0830***	0.0075	0.057
Curvilinear (BMI, BMI^2^)	0.2711, −0.0037	0.3503, −0.0066	0.720, 0.956	0.0949, −0.0002	−0.1119, 0.0021	0.089, 0.128
Face						
Linear (BMI)	−0.0272*	−0.0362**	0.831	0.0177	0.0000	0.817
Curvilinear (BMI, BMI^2^)	−0.3113*, 0.0049*	0.0777, −0.0020	0.086, 0.075	0.0034, 0.0002	0.3253, −0.0055	0.544, 0.498
Thorax						
Linear (BMI)	0.0613****	0.0477**	0.486	0.0572**	0.0419**	0.446
Curvilinear (BMI, BMI^2^)	0.3134*, −0.0043	0.1853, −0.0024	0.561, 0.609	−0.1929, 0.0041	0.0425, −0.0000	0.514, 0.477
Abdomen						
Linear (BMI)	0.0317	0.0603***	0.804	−0.0578	−0.0182	0.338
Curvilinear (BMI, BMI^2^)	0.0631, −0.0005	0.3898*, −0.0056	0.611, 0.578	−0.6144**, 0.0097**	−0.6251*, 0.0106*	0.663, 0.755
Spine						
Linear (BMI)	0.0585***	−0.0117	0.061	0.0454	0.0255	0.604
Curvilinear (BMI, BMI^2^)	0.3868, −0.0055	0.0495, −0.0011	0.322, 0.384	−0.2983, 0.0056*	0.5442, −0.0093	0.264, 0.249
Upper extremity						
Linear (BMI)	−0.0178	0.0112	0.113	0.0353	0.0429*	0.913
Curvilinear (BMI, BMI^2^)	−0.2114, 0.0033	−0.1407, 0.0027	0.452, 0.582	0.3774, −0.0057*	0.4955**, −0.0078**	0.764, 0.674
Lower extremity						
Linear (BMI)	0.0103	0.0752****	0.036	0.0214	0.0285	0.944
Curvilinear (BMI, BMI^2^)	−0.2709, 0.0048*	0.2439, −0.0029	0.169, 0.203	−0.2720*, 0.0049**	−0.0471, 0.0013	0.383, 0.415
**ΔV Model**						
Head						
Linear (BMI)	0.1144***	0.0106	0.070	0.1436^e^ ***	−0.0776^e^	0.0005^e^
Curvilinear (BMI, BMI^2^)	0.1175, −0.0001	0.3838*, −0.0066**	0.914, 0.643	0.0062, 0.0023^e^	−0.5288*, 0.0081^e^	0.072, 0.180^e^
Face						
Linear (BMI)	0.0070	−0.0313***	0.129	0.1602^f^ **	0.0897^f^ *	0.297^f^
Curvilinear (BMI, BMI^2^)	−0.2454**, 0.0044**	0.0727, −0.0018	0.021, 0.015	0.2660, −0.0017^f^	2.379, −0.0371^f^	0.161, 0.126^f^
Thorax						
Linear (BMI)	0.1168****	0.0529***	0.007	0.1622***	0.0071	0.0002
Curvilinear (BMI, BMI^2^)	0.4630**, −0.0059*	0.0946, −0.0007	0.220, 0.298	0.0265, 0.0022	−0.1427, 0.0026	0.154, 0.395
Abdomen						
Linear (BMI)	0.1357****	0.0691****	0.041	−0.0689^g^ **	−0.1292^g^ **	0.093^g^
Curvilinear (BMI, BMI^2^)	0.6316***, −0.0083**	0.6169**, −0.0093*	0.728, 0.966	−0.7044***, 0.0113^g^ **	−1.3421****, 0.0219^g^ ****	0.178, 0.183^g^
Spine						
Linear (BMI)	0.0821***	−0.0094	0.060	0.1981**	−0.0285	0.021
Curvilinear (BMI, BMI^2^)	0.6899**, −0.0104*	0.0828, −0.0016	0.171, 0.202	−0.4873, 0.0108	−0.1236, 0.0017	0.610, 0.469
Upper extremity						
Linear (BMI)	−0.0196	−0.0081	0.890	0.0628	0.0027	0.349
Curvilinear (BMI, BMI^2^)	−0.2750*, 0.0045	−0.0761, 0.0012	0.337, 0.304	0.7580, −0.0119	0.1509, −0.0026	0.119, 0.134
Lower extremity						
Linear (BMI)	0.0396*	0.0591***	0.473	0.0376	−0.0092	0.328
Curvilinear (BMI, BMI^2^)	−0.1296, 0.0029	0.3077, −0.0043	0.228, 0.237	−0.4702**, 0.0086**	−0.1656, 0.0027	0.210, 0.147

The analysis was limited to drivers who were involved in frontal collisions only. ΔV was not included in all subjects models but was included in the ΔV model, so the subjects with ΔV not available were excluded during the analysis for the ΔV model. Two outcomes were used (see note a), and the risk factor was BMI, represented by linear (BMI) and quadratic (BMI^2^) terms. Covariates in the model were age, race, alcohol involvement, drug involvement, type of vehicle, vehicle age, curb weight, seat belt use, air bag deployed, ejection, rollover, type of collision, manner of collision, road speed limit, light condition, weather, and ΔV (for ΔV model); sex was included in the pooled model. All categorical variables were processed as dummy variables.

a Two cutoffs of regional body maximum AIS were used to define regional body injury severity outcomes: “injured or not injured” was defined by max AIS equal to 0 or not (injured: max AIS >0; not injured: max AIS  = 0); “seriously injured versus not seriously injured” was defined by max AIS equal to 3 or over (seriously injured: max AIS ≥3; not seriously injured: max AIS<3). Sex difference was evaluated in the pooled model by adding interactions between sex and risk factors in the model and the two sexes were analyzed by logistic regression models separately.

b Coefficients from the logistic regression models are presented, and the stars attached to the coefficients show statistical significance.

c Interactions between BMI (and BMI^2^) and sex were tested in the pooled model and the *p-*values of these interactions are presented.

d The number of serious head injuries in some subgroups of seat belt use and airbag deployment was too small to estimate the regression model, so these two variables were omitted in the model.

e The number of serious face injuries in some subgroups of airbag deployment and ejection was too small to estimate the regression model, so these two variables were omitted in the model.

f The number of serious abdomen injuries in some subgroups of seat belt use and airbag deployment was too small to estimate the regression model, so these two variables were omitted in the model.

*, *p*<0.10.

**, *p*<0.05.

***, *p*<0.01.

****, *p*<0.0003 (Bonferroni corrected significance).

**Figure 1:**
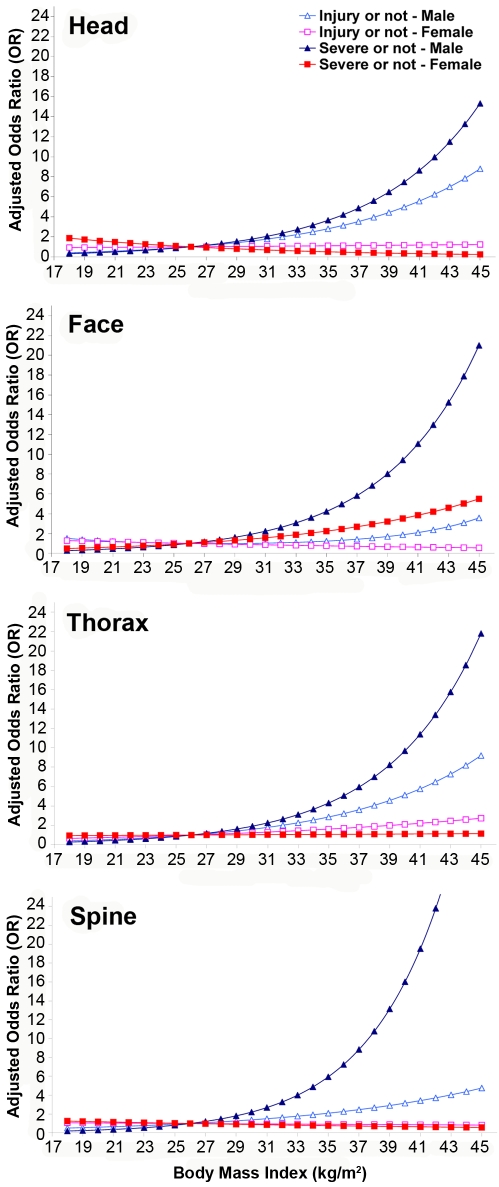
Adjusted OR for being injured or seriously injured by drivers' BMI and sex in upper body regions: head, face, thorax, and spine. ΔV model, frontal collision only, *n* = 3,491 (male model, weighted size: 1,522,221), *n* = 2,440 (female model, weighted size: 1,142,701), and *n* = 5,931 (pooled model, weighted size: 2,664,922).

**Figure 2:**
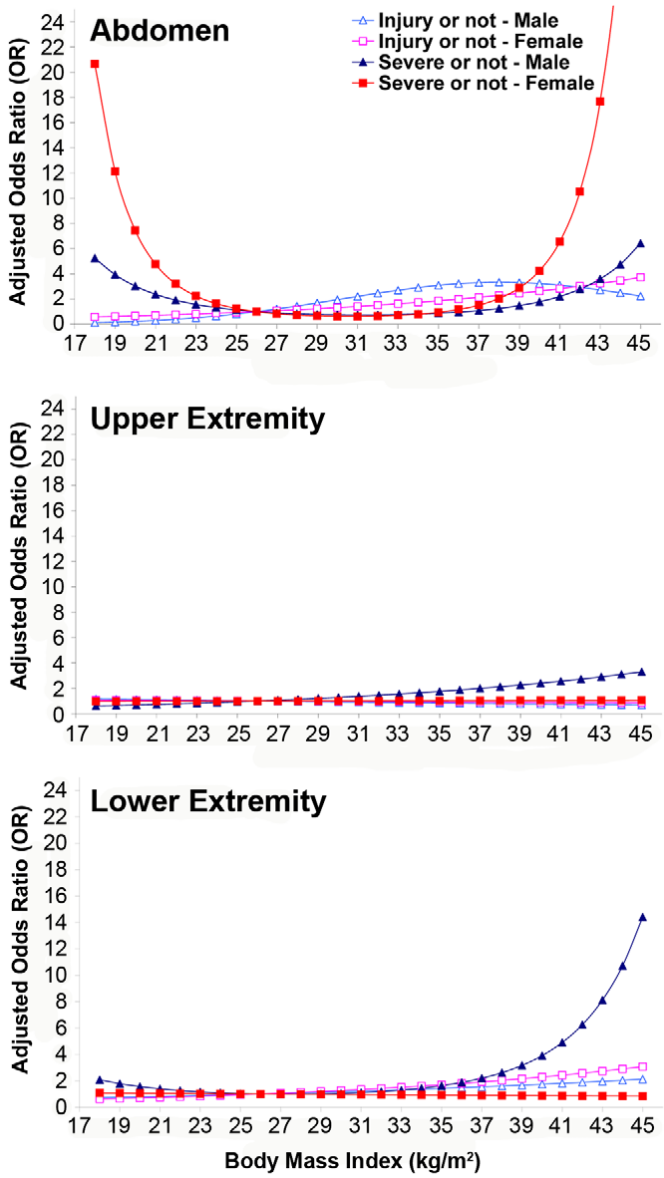
Adjusted OR for being injured or seriously injured by drivers' BMI and sex in the abdomen, upper extremity, and lower extremity. ΔV model, frontal collision only, *n* = 3,491 (male model, weighted size: 1,522,221), *n* = 2,440 (female model, weighted size: 1,142,701), and *n* = 5,931 (pooled model, weighted size: 2,664,922).

The adjusted ORs derived from the logistic regression in the ΔV model of the BMI continuum for head, face, thorax, and spine injuries are shown in Figure 1. The adjusted ORs for the abdomen, upper extremity, and lower extremity are shown in Figure 2. Compared to nonobese male drivers, obese male drivers had a higher risk for injury and serious injury to the head, thorax, and spine. Male drivers had a higher risk of being injured in the head, thorax, and spine for both outcomes, injured versus not injured and seriously injured versus not seriously injured, than did female drivers. In addition, a *U*-shaped relation between BMI and serious injury in the abdominal region was found for both male and female drivers.

Information was missing on ΔV for approximately 44% of drivers. Drivers' age, BMI, and proportions of race-ethnicities did not differ significantly between the groups for which ΔV was available or not. A higher proportion of subjects in the ΔV group were injured in the thorax, spine, and upper and lower extremities, and a lower proportion of subjects were seriously injured in the spine. The interaction terms between ΔV group and BMI (or BMI and BMI^2^) were not significant except for abdomen and upper extremity injuries for the injured versus not injured outcome and for face injury for the seriously injured versus not seriously injured outcome.

**Figure 3:**
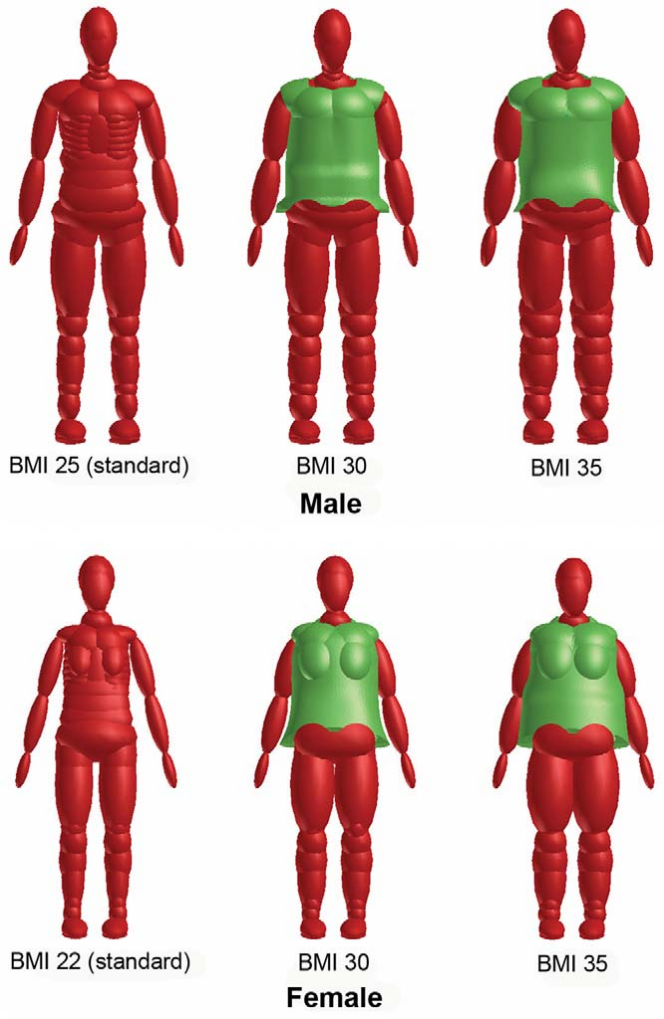
Standard and obese dummy models for male (top) and female (bottom).

Figure 3 illustrates the standard and obese dummies used in the model simulations for male and female. All of the male dummies have the same height as 1.77 m. The weights of the male dummies are 78.4, 92.7, and 110.0 kg for the BMI 25, 30, and 35 models, respectively. The weight of torso subcutaneous adipose tissue was 9.4 kg for the BMI 30 model and 13.5 kg for BMI 35 model. All of the female dummies have the same height of 1.52 m. The weights of the female dummies are 49.3, 69.4, and 80.8 kg for the BMI 22, 30, and 35 models, respectively. The weight of torso subcutaneous adipose tissue was 14.3 kg for the BMI 30 model and 17.2 kg for BMI 35 model.

**Figure 4:**
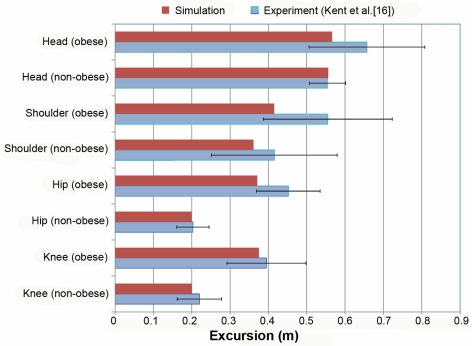
Comparison of head, shoulder, hip, and knee excursions between model simulation and experiment [16].

Figure 4 shows the result of model validation study. The body kinematics of standard and obese dummies was consistent with that of PMHS subjects. Even though the body excursion of the obese dummy was predicted to be less than that of the PMHS subjects due to dummy's lighter weight (110 kg) than the subjects (mean weight 128 kg) used in the experiment, the decreased torso pitch induced by increased hip excursion of the obese subjects was well predicted in the model simulation. The ratios of shoulder excursion to hip excursion were 1.12 (simulation) and 1.23±0.37 (experiment) for obese subjects, and 1.82 (simulation) and 2.05±0.80 (experiment) for nonobese subjects.

**Figure 5:**
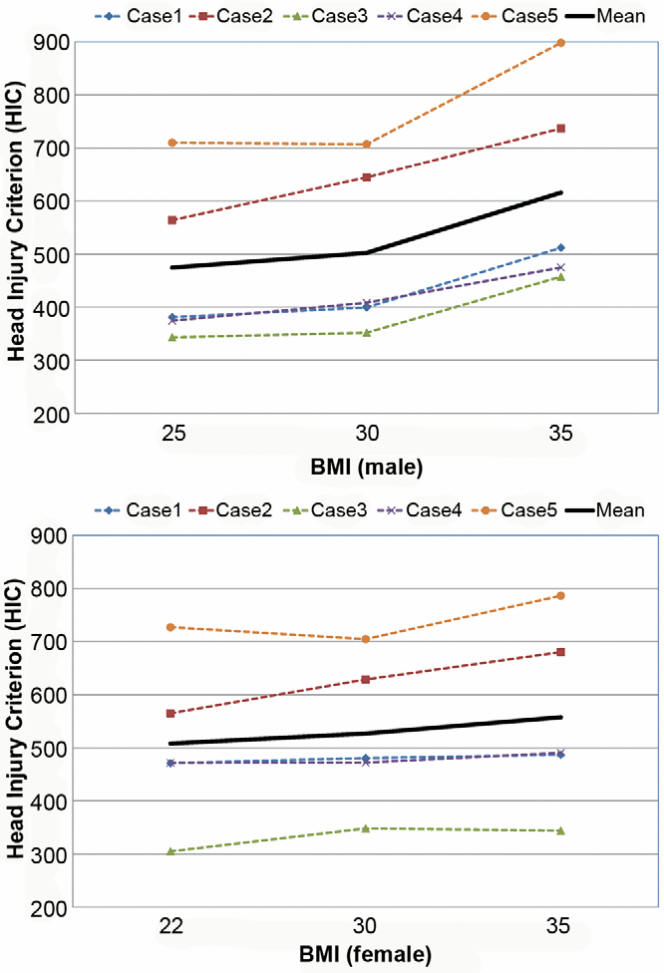
Computational investigation of the effect of obesity on the head injury criterion (HIC) for male (top) and female (bottom).

Videos S1 and S2 are the animated results of the model simulation to compare the kinematics of the standard and obese dummies (BMI 35) during a frontal crash (Case 1) for male and female, respectively. From the simulation outcomes, the regional body injuries (head injury criterion [HIC], neck injury criterion [Nij], thorax, and lower extremity criterion [LEC]), which are based on the mechanical responses (regional acceleration, force, moment, deflection) from the six different dummies shown in Figure 3, were measured for each simulation case (total five cases). Figures 5–9 show the variations of the injury measures as BMI increases, for head, neck, thorax (chest acceleration and deflection), and lower extremity, respectively. From the results, obese males have a much increased risk of injury (especially head, chest acceleration, chest deflection, and lower extremity) as compared to the standard male. Meanwhile, obese females have much increased (chest acceleration), slightly increased (head and lower extremity), or decreased (neck and chest deflection) risk of injury as compared to the standard female.

**Figure 6 pmed-1000250-g006:**
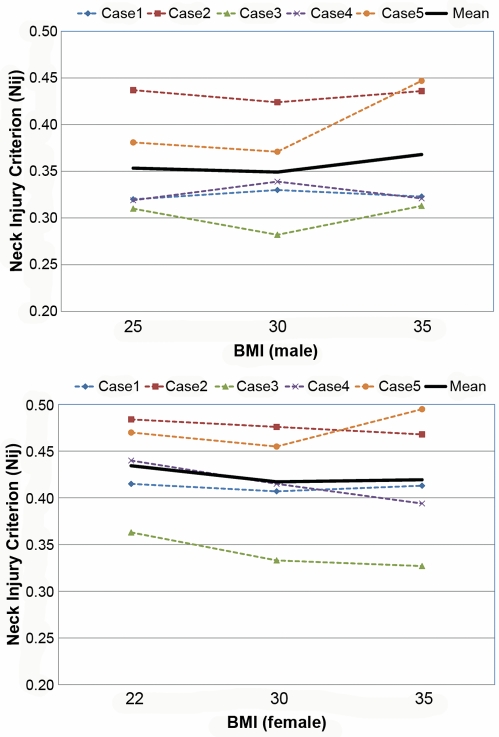
Computational investigation of the effect of obesity on the neck injury criterion (Nij) for male (top) and female (bottom).

**Figure 7 pmed-1000250-g007:**
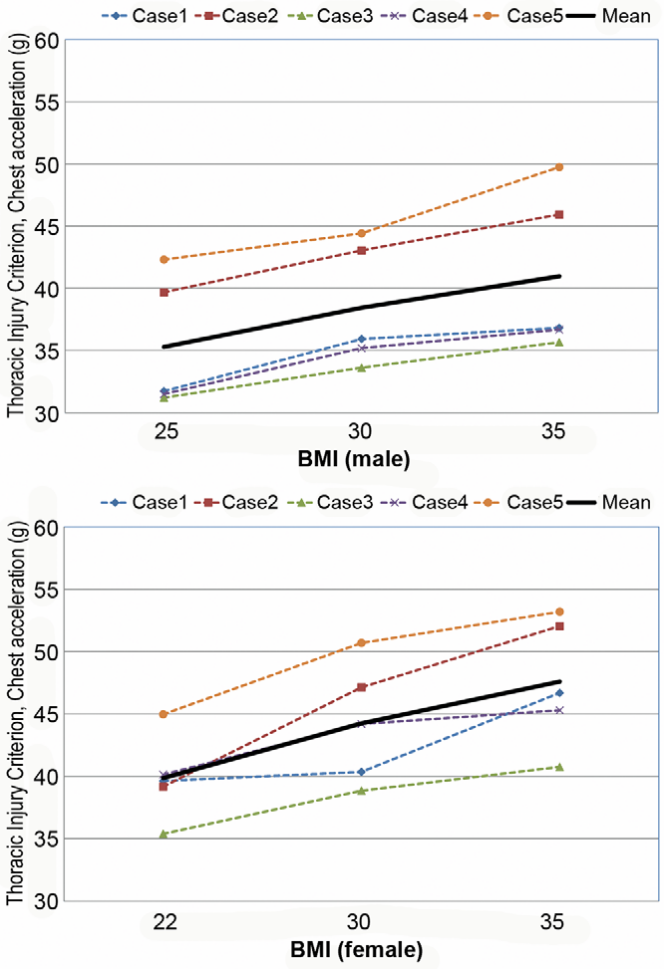
Computational investigation of the effect of obesity on the thorax injury criterion (chest acceleration, g) for male (top) and female (bottom).

**Figure 8 pmed-1000250-g008:**
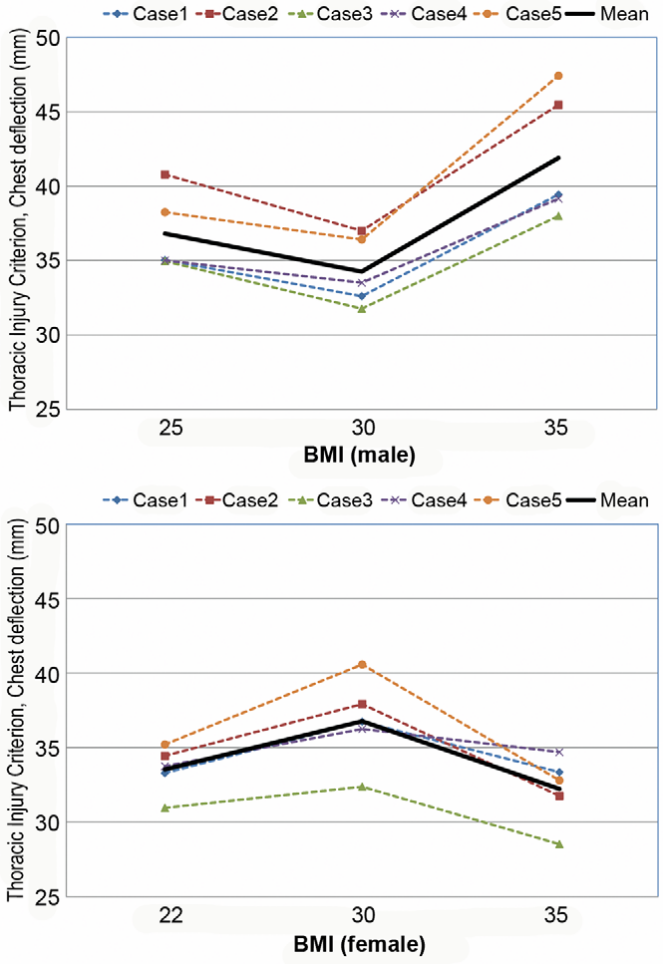
Computational investigation of the effect of obesity on the thorax injury criterion (chest deflection, mm) for male (top) and female (bottom).

**Figure 9:**
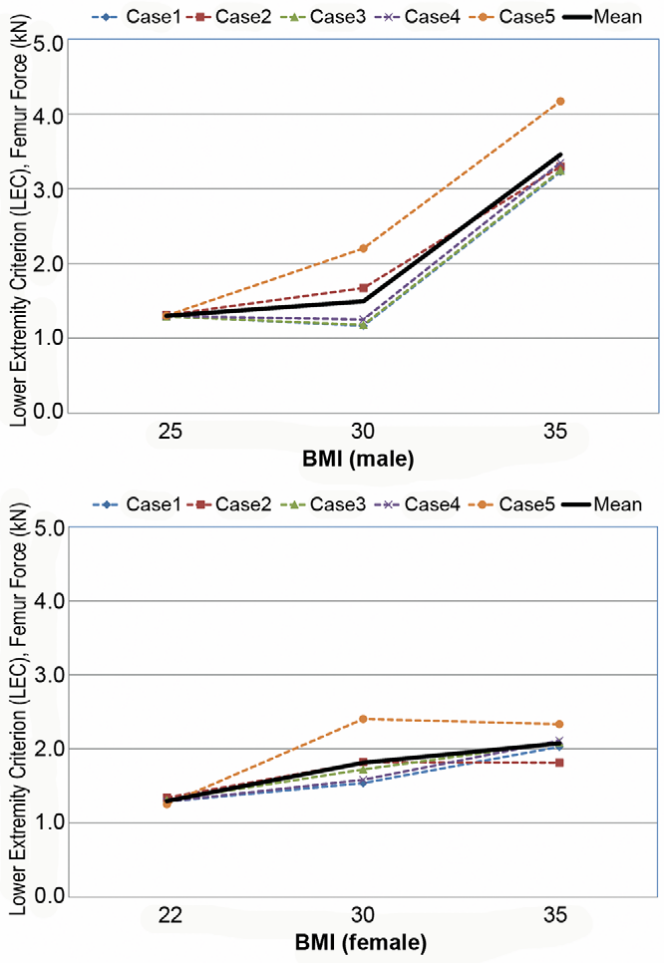
Computational investigation of the effect of obesity on the lower extremity injury criterion (LEC) for male (top) and female (bottom).

According to the results of sensitivity analysis, no factors showed a significant impact on the models except for ΔV; however, crashes with ΔV available have been analyzed separately in our study. Results for belted and unbelted drivers were calculated separately and compared to the results for all drivers. Both belted and unbelted drivers showed very similar injury patterns with the results of both combined, especially for upper body regions, in all outcome models that could be estimated successfully (101 out of 112 models). In the other 11 cases (for example, serious facial injury in the all-subjects model for females, serious upper extremity injury in the ΔV models for both sexes), sample sizes in some covariate combinations were insufficient to estimate the models. Given this robust experience with sensitivity analysis, we have reported results for combined analyses that retain seat belt use as an indicator covariate in all models.

## Discussion

This study used both real-world and computer crash simulation data to examine injury patterns in MVCs, associations between BMI and regional body injury, and the differences in these associations by sex. From the real-world data, we found that obese male drivers have a substantially higher risk for injury, especially serious injury, to the upper body regions of the head, face, thorax, and spine. Obese female drivers, by contrast, have a slightly increased risk for injury to the thorax, abdomen, and lower extremities but a decreased risk for injury to the face. In addition, we found a *U*-shaped relation between BMI and serious injury in the abdominal body region for both men and women. The sex difference in the association between BMI and risk of injury and serious injury was found in head, thorax, and spine. In the high BMI range, male drivers were more likely to be seriously injured than were female drivers for all body regions except for extremities and abdominal region. The computer simulation crash test results confirmed the findings from real-world observations showing that obese men experienced a higher risk of upper body injury than did nonobese men.

The associations between obesity and regional body injury are not well understood. In the present study, these associations are identified and we find that obese male drivers have a higher risk for injury and serious injury to the head, face, thorax, and spine than do nonobese male drivers. The findings were also confirmed by computer simulations of crash tests in this study. This finding of a higher risk of head injury for obese male drivers is consistent with an earlier study that was based on the same database [20]. However, the study population in that study was passengers and both male and female passengers were pooled together, thus different injury patterns for two sexes were not addressed in the study. The findings of the present study differ from other previous studies. Studies conducted by Boulanger et al. [21] and Moran et al. [22] found that obese patients suffered less head injury in MVCs than did nonobese patients. Those authors explained that these fewer head injuries were because of the small head/torso mass ratio of obese persons and because the torso could sustain the crash impact and spare the head from injury. The different results in the present study might be due to differences in the definitions of obesity and health outcomes and the databases used. Our findings for thoracic injury confirm the results of previous studies in which obese drivers had a greater risk of injury than did nonobese drivers [21]–[23].

Both obese men and obese women showed an increased risk of abdominal injury in the present study, which contrasts with the results of previous studies [21],[22],[24]. However, for the risk of serious abdominal injury, the current results confirm the previous results that overweight (25≤BMI<30) persons have a lower risk, but obese (BMI ≥30) persons have a much higher risk than the nonobese. For obese persons, the increase in energy transfer and momentum might override this protection effect and cause more severe injury [24].

Obese male drivers had a higher risk of injury in all upper body regions, including the head, face, thorax, abdomen, and spine, than did obese female drivers. For serious injury, the risk was higher in obese male drivers than in female drivers in the upper body regions of the head, thorax, and spine. The reasons for these differences are still unknown; however, the body fat distribution, body shape, and center of gravity may play an important role in the different injury patterns and severity of injury between men and women. In our analysis of the center of gravity with use of MRI technology, we found that men have a higher center of gravity than do women, especially among the obese [25]. A higher center of gravity interacting with a change of velocity might cause a greater force toward the upper body regions in male drivers.

The higher risk of injury for male drivers associated with a high BMI could be caused by some combination of momentum effects, comorbidities of obesity, and the body's response to crash and injury as the result of the anatomical and physiological changes with obesity [23],[26]–[30]. Current vehicle cabin design is in accordance with the Federal Motor Vehicle Safety Standard that used the 50th percentile male Hybrid III Crash Dummy (H3CD, 1.78 m, 77.11 kg in the driver's position, BMI = 24.3 kg/m^2^) [22]. These cabin designs may not be optimal for drivers whose body size and shape differ considerably from the standard specifications and may contribute to an increased risk of injury for persons in the high BMI ranges [31],[32].

Socioeconomic status (SES) has been associated with obesity, thus the different pattern of crash and injury severity between obese and nonobese people may result from SES inequality. In this study, we have adjusted several SES-related variables, such as race/ethnicity, vehicle age, vehicle type, and vehicle curb weight. However, most SES variables, such as education, income, and occupation are not available in NASS CDS database. In the current study, sex difference was found for the pattern of regional body injury between men and women. SES should have a similar effect on injury pattern for both genders after controlling vehicle-related variables, thus this sex difference of body regional injury could not be explained by possible SES inequality. Several previous studies have demonstrated the role of SES on the severity of MVCs [33]–[35]. During the analyses in this study, the variables related to the severity of MVCs, such as the change in velocity (ΔV) and type of collision were adjusted; however, the obese people experienced more severe outcomes even when controlling for severity of crashes.

The results of our study should be interpreted in light of several limitations and strengths. Approximately 19.6% of the drivers who were eligible for the study were subsequently excluded from the analyses. Missing data was the leading cause of these exclusions and might have caused bias in either direction in the associations found in both sexes. However, it was not likely that such exclusions affected the results beyond decreasing the precision of the statistical estimates [22]. Bias may have occurred in the ΔV model, because ΔV information was not available for 44.6% of the drivers. Our additional analyses, however, indicated no significant differences in demographic variables between the groups for which ΔV information was available or not. Significant differences were found in some collision and environment-related variables, which may have been because of the difficulties of measuring ΔV in some kinds of collisions and environments [8]. Our analyses were restricted to frontal collisions, which may have somewhat reduced the effects of inaccuracies on ΔV measurements. Because of the lack of comorbidity data in the NASS CDS, comorbidity was not included in the models. Baseline illness such as vision impairment was not controlled in our analysis because it was not available in the database. Sitting height may be more appropriate as an anthropometry index for occupants sitting in the vehicles; however, it was not available in the database. A potential limitation of the computational study is that the hybrid III ellipsoidal dummy models were used as baseline models. Even though the body kinematics was well predicted with those models, details of biomechanical responses (i.e., regional stress and strain) of bone, joint, and tissue could not be obtained. Future efforts could go towards a more robust validation and simulation with more “human-like” models such as the facet or finite element model, when fully validated models for both male and female are available.

Our study had several strengths. This study examined the patterns of associations between BMI and regional body injury severity and the differences in these associations by sex. Our study integrated both real-world data and computer simulation crash tests designed to evaluate regional body injuries during MVC. The findings of the present study may contribute to our understanding of the potential mechanism of sex-specific effect of obesity on MVC injury. If the mechanisms can be established, corresponding applications to vehicle safety designs could be customized and provided to obese people for better protection. All potential covariates and confounders from literature and our previous studies were included in our regression models, and simultaneously adjusting for these covariates and confounders allowed us to identify the correlation between BMI and regional body injury severity over and above these factors. The large number of observations in the NASS CDS dataset used enabled us to describe the associations between BMI and regional body injury at an individual level with a high degree of statistical precision. We focused on frontal collisions and used separate analyses for men and women to eliminate potential differences in sex, causal pathways, and confounding factors between drivers and passengers among different collision types.

### Conclusions

Our study identified the relation of BMI to regional body injuries and their severity using both real-world and computer crash simulation data. Our findings are consistent with the previously observed BMI-related fatality risk profile. This observation implies that mechanical factors may be implicated in this risk. Such factors, if further elucidated, can be addressed by motor vehicle design changes. In addition, the observed sex differences may be related to differences in body shape, center of gravity, and adipose tissue distribution. A more precise connection between these factors cannot be made at this time without further investigations into the biomechanical responses of the human body considering a variety of dummy models configurations with different height and weight, various crash acceleration/deceleration pulses and occupant restraint systems, and other aspects that may lead to injury. Nonetheless, our findings may have important implications for high-risk cohort identification (e.g., obese male drivers), traffic safety intervention, policymaking, and for motor vehicle design to protect more vulnerable body regions.

## Supporting Information

Video S1 Animated results of Case 1 for male with BMI 25 dummy (left) and BMI 35 dummy (right).
(9.05 MB AVI)

Video S2 Animated results of Case 1 for female with BMI 22 dummy (left) and BMI 35 dummy (right).
(8.75 MB AVI)

## Abbreviations

AIS: abbreviated injury scale
BMI: body mass index
CI: confidence interval
CDS: crashworthiness data system
MADYMO: mathematical dynamic models
MVC: motor vehicle crash
NASS: national automotive sampling system
OR: odds ratio
PMHS: postmortem human surrogates
SES: socioeconomic status
